# Open mitral comissurothomy in surgery of isolated mitral stenoses

**DOI:** 10.1186/1749-8090-8-S1-O289

**Published:** 2013-09-11

**Authors:** V Popov, V Lazoryshinets, V Shimon, V Mnishenko

**Affiliations:** 1 Department of Acquired Heart Diseases, National Amosov's Institute of Cardiovascular Surgery, Kyiv, Ukraine

## Backgound

To research possibilities of open mitral comissurothomy (OMC) in correction of mitral valve disease (MVD).

## Methods

Analyzed group consists of 225 patients (pts) with MVD operated in Institute from 01.01.1081 till 01.01.2008. Average age was 51,3±6,1 yy. 151 (67,1%) pts belonged to IV NYHA class, 69 (30,7%) – to III and 5 (2,2%) pts to II. There were 69 (30,7%) males and 156 (69,3%) females. Mitral stenosis was in 191(84,9%) pts, combined MVD in 34 (15,1%) pts. Isolated OMC was performed In 146 (64,9%) pts, in 79 (35,1%) cases – in combination with other plastic procedures on MV including ring annuloplasty, suture comissuroplasty, resection of posterior leaflet. Thrombectomy of left atrium was performed in 71 (31,6%) pts including 21(9,3%) pts with massive thrombus formation. 9 (4,0%) pts were cerebral cysts after previous episods of thromboemboli. Tricuspid valve correction was occured in 69 (30,7%) pts All operations were performed with cardiopulmonary bypass, moderate hypothermia and crystalloid cardioplegia.

## Results

Among 225 operated pts on hospital period (30 days after operation) 2 pts died (hospital mortality - 0,9%). Causes of deaths: pneumonia (n=1), brain damage (n=1). Transient neurological complications were in 3 (1,3%) pts. During echocardiography on hospital stage gradient on MV was 5,8±0,7 mm Hg, regurgitation 1+ marked in 13 (5,8%) cases. In remote period 13,8±1,7 yy good-satisfactory result was marked in 89,4% pts. 5 pts (2,3%) died. Reoperations were performed in 9 (4,1%) cases.

## Conclusion

OMC is adequate method of surgical correction of MVD with minimal risk of developing of fatal complications. It is acceptable as isolated and in addition with ring annuloplasty, suture comissuroplasty. In case of small cavity of left ventricle OMC may be alternative to prosthesis, allows to exclude obstruction of outlet of left ventricle, and in pts with massive thromboses of LA to decrease level of thromboembolic events at remote period.

